# Comparative analysis of tear cytokines in patients with glaucoma, ocular hypertension, and healthy controls

**DOI:** 10.1007/s10792-023-02763-6

**Published:** 2023-06-15

**Authors:** Dominika Mravec Bencurova, Petr Vyborny, Pavlina Dankova

**Affiliations:** 1 Department of Anthropology and Human Genetics, Faculty of Science, Charles University, Viničná 7, 12843 Prague 2, Czech Republic; 2 Eye Department, 1-St Faculty of Medicine, Charles University and Central Military Hospital Prague, Prague 6, Czech Republic

**Keywords:** Glaucoma, Ocular hypertension, Tears, Cytokines, Inflammation

## Abstract

**Purpose:**

To investigate the ocular surface inflammation in patients with primary open angle glaucoma and ocular hypertension by analyzing tears and to compare findings with healthy controls.

**Methods:**

Observational case–control study. Tear samples were collected by 5 µl microcapillary tube from 24 patients with glaucoma treated by antiglaucoma drops, 9 non-treated patients with ocular hypertension and 45 healthy controls. Tears were analyzed from right eye by multiplex Bio-Plex system for the presence of 6 cytokines: IL1β, IL10, IL4, IFNγ, MIF and VEGF.

**Results:**

Significantly higher concentrations of IL1β and IL10 (glaucoma or ocular hypertension vs. healthy controls, *p* < 0.0001), VEGF (glaucoma vs. ocular hypertension, *p* < 0.05; ocular hypertension vs. healthy controls, *p* < 0.02) and MIF (glaucoma vs. healthy controls, *p* < 0.03) were detected in patients’ tears. Both patient groups have activated to a significantly lower extent the Th1 pathway represented by IFNγ than Th2 pathway represented by IL10 (*p* < 0.001) and, at the same time, the IFNγ/IL4 ratio was significantly increased in healthy controls (*p* < 0.001) and patients with ocular hypertension (*p* < 0.02) compared to glaucoma individuals.

**Conclusion:**

This study shows that secretion of inflammation-related cytokines by conjunctival cells is increased in both, glaucoma and ocular hypertension patients and can be detected in their tears. Nevertheless, data indicates stronger ocular surface inflammation in non-treated follow-up patients diagnosed with ocular hypertension than in glaucoma subjects treated by antiglaucoma drops.

## Introduction

Primary open angle glaucoma (POAG) is a multifactorial neurodegenerative disease with unclear pathogenesis, making early diagnosis the main challenge. This eye disease affects retinal ganglion cells and, due to this, is also one of the main causes of irreversible blindness worldwide [1].

An important parameter in the diagnosis is the magnitude of intraocular pressure (IOP). The IOP is influenced by various external and internal factors including the time within the day [2] and the year, heartbeat [3], respiration [4] and nutrition [5, 6]. The existence of the true causal relationship has been discovered in the 1990s by showing that lowering IOP can slow down or even prevent POAG progression [7–10]. Notably, some patients with POAG have normal IOP; therefore, in addition to IOP measurement, diagnosis of glaucoma is also based on examination of the optic disk, determination of the retinal nerve fiber layer (RNFL) and evaluation of visual functions. Currently, another potential diagnostic and prognostic possibility is the examination of biomarkers in tears. Biomarkers can serve not only for exact POAG diagnosis; they may also help identify subjects at increased risk of developing POAG. Biomarkers for eye disease are sought and studied in the aqueous humor [11], trabecular meshwork [12], optic nerve [13], blood [14] and recently also in tears [15].

Tear sampling represents a convenient and noninvasive method of analyzing an accessible body fluid for the investigation of disease-specific biomarkers in local environment. Tears are a complex fluid that contains thousands of molecules, including cytokines [16]. In the past, the quantification of cytokines in tears was limited by the small volume of tears available per sample. Currently, cytometric multiplex bead analysis can determine the multiple cytokine profile in the volume of no more than 5 µl tear sample [17, 18].

In glaucoma, the most interesting may be indicators of inflammation, apoptosis, or other types of cell death that could reveal the general condition of the ocular surface, either as a response to treatment, exogenous/endogenous influences, or to monitor disease progression. The ambition of the GRIM study (Glaucoma—role of immunity and inflammation) conducted at the Central Military University Hospital Prague and the Faculty of Science of Charles University, Prague, Czech Republic, is to analyze 6 cytokines (interleukin (IL)1β, IL4, IL10, interferon gamma (IFNγ), macrophage migration inhibitory factor (MIF) and vascular endothelial growth factor (VEGF)) in tears of patients with ocular hypertension (OHT) or primary open-angle glaucoma (POAG) that could be involved in immunoinflammatory, apoptotic, and proliferative responses to treatment, neuroinflammation, elevated IOP and RNFL thickness. Examined cytokines cover both, Th1 and Th2 immune responses. We aim to find out if concentrations of selected cytokines in tears change in relation to these characteristics similarly as observed in aqueous humor [11], a trabecular meshwork [12], or in retinal cells [13]. Of particular interest are the potential differences between POAG and OHT patients. To answer the question whether glaucoma-specific changes in cytokine optic nerve affect the concentrations of cytokines in tears the Bio-Plex multiplex system has been used.

## Materials and methods

### Patients, controls and sample collection

Samples were obtained from 24 medicated patients (12 males, 12 females) with open angle glaucoma, and 9 patients diagnosed with ocular hypertension (4 males, 5 females) at the Central Military University Hospital Prague. Forty-five volunteers (24 males, 21 females) with no history of ocular surface disorders were employed as healthy controls (HC). Written consent and questionnaire about health, medical history and lifestyle were obtained from all subjects prior to their participation. The study adhered to the principles of the Declaration of Helsinki and was approved by the Ethics Committee of the Faculty of Science, Charles University (Institutional Review Board, Faculty of Science, Charles University, Prague, Czech Republic) and by the Ethics Committee of the Central Military University Hospital (Institutional Review Board, Central Military University Hospital, Prague, Czech Republic). Patients with diabetes, any autoimmune or eye disease other than POAG or OHT including dry eye syndrome were excluded. Patients were diagnosed as the POAG or OHT based on the IOP level, optic disk and visual field examination. In the present study, patients with IOP above 25 mmHg at the time of OHT diagnosis but without morphological and functional changes were assigned to the OHT group. Clinical data such as the magnitude of intraocular pressure on the day of tear collection, previous eye disease record or laser refractive surgery, spherical equivalent refractive error (between − 1.0 and + 1.0 diopter), results from optical coherence tomography (OCT) presented as RNFL thickness value, perimeter and glaucoma medication were available for all patients. Tear samples were collected without topical anesthesia, from the inferior fornix using a 5 µl capillary tube. Tears were expelled from the capillary tube into a 1 ml collection tube and diluted in 150 µl of phosphate-buffered saline (final volume 155 µl). After dilution, the tears were stored at − 80 °C until further processing.

### Multiplex cytokine analysis

The Bio-Plex multiplex system (Bio-Rad Laboratories) was used to determine the levels of 6 cytokines in tears: IL1β, IL4, IL10, IFNγ, VEGF and MIF. This system uses a liquid suspension array of 6 sets of 5.5 um magnetic beads internally labeled with different ratios of two spectrally distinct fluorochromes to assign a unique spectral address. Each set of beads was combined with a monoclonal antibody against IL1β, IL4, IL10, IFNγ, VEGF and MIF, respectively. Each sample was measured in duplicate. Standard curves were generated by using the reference cytokine concentrations supplied by the manufacturer. The raw data were analyzed by Bio-Plex Manager Software (Bio-Rad Laboratories) to obtain concentration values; data that did not meet the accuracy and precision criteria were excluded from subsequent analyzes in accordance with the manufacturer’s recommendation.

### Statistical analysis

Data were analyzed using GraphPad Prism 6 software. The D’Agostino-Pearson normality test was used to check normality. Statistical significance for intergroup differences was assessed by the nonparametric Wilcoxon signed rank test and the Mann–Whitney *U* test. The level of statistical significance was established at *p* < 0.05. The clinical characteristics are presented as mean and SEM (standard error of mean) (Tab. 1); the immunological data are presented as median with the interquartile range.

**Table 1:** Clinical characteristics of subjects

Characteristics	HC	POAG	OHT	*p* value
	(*n* = 45)	(*n* = 24)	(*n* = 9)	
Male	24	12	4	
Female	21	12	5	
Age [years] (mean; SEM)	34.7; 1.6	44.6; 1.8	36.1; 3.1	*p* = 0.0006
Duration of treatment [years] (mean; SEM)	NAP	6; 0.7	NAP	NA
Ocular pressure [mmHg] (mean; SEM)	NA	17; 0.75	20; 1.36	*p* = 0.04
Prostaglandin analogs	0	14	0	
Timolol and prostaglandin analogs	0	3	0	
Timolol	0	4	0	
Beta-blockers	0	1	0	
Without treatment	45	2	9	

HC, healthy controls; POAG, patients with open angle glaucoma; OHT, patients with ocular hypertension; NA, not available; NAP, not applicable; SEM, standard error of mean; n, total number of subjects

## Results

### Subjects

Table 1 displays the characteristics of subjects participating in the study: 24 patients with POAG, 9 patients with OHT and 45 healthy controls. The most patients with POAG were treated more than 1 year and less than 12 years with antiglaucoma medication.

### Concentration of cytokines in tears 

**Fig. 1:**
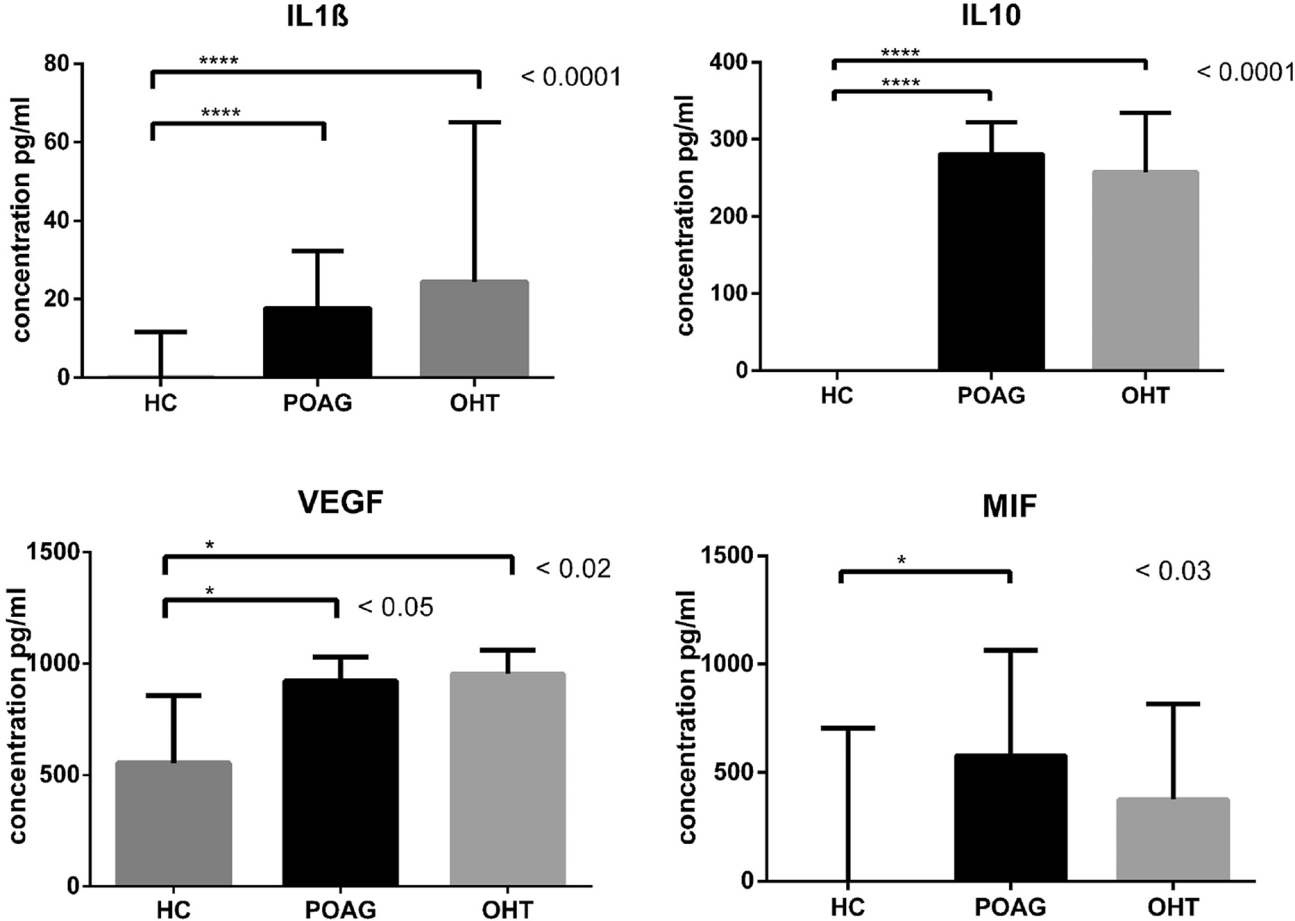
Concentrations of IL1β, IL10, VEGF and MIF cytokines (pg/ml) in tears derived from healthy controls (HC) and patients with glaucoma (POAG) or ocular hypertension (OHT). Columns represent the median of cytokine concentration of all individuals; error bars depict group median with interquartile range. Four asterisks denote statistically significant difference *p* < 0.0001, one asterisk denotes statistically significant difference *p* < 0.05

The concentrations of IL1β, IL10 and MIF cytokines in tears from the HC subjects were below the detection limit in the most samples. Compared to them, we observed significantly higher concentrations of IL1β in tears from patients with POAG (*p* < 0.0001) or OHT (*p* < 0.0001) and similarly, IL10 levels in both POAG and OHT tears were significantly amplified when compared to HC (*p* < 0.0001; please see Fig. 1). Further, significantly higher concentrations of MIF cytokine in tears from POAG patients compared to HC individuals were observed (*p* < 0.03). VEGF levels in tears of both patient groups were increased in comparison to those in tears from healthy controls (POAG *p* < 0.05, OHT *p* < 0.02; please see Fig. 1).

Comparison between the two diseased groups revealed insignificant 1.4fold increase of IL1β and IFNγ levels and conversely, 1.5times lower concentrations of MIF cytokine in tears from OHT patients (all *p* = NS). The summary of detection limit of each cytokine, percentage representation of subjects below the detection limit and median with interquartile range for each group and each cytokine are displayed in Table 2.

**Table 2:** Detection limit, median and interquartile range of analyzed cytokines

Cytokine	Detection limit [pg/ml]	% Samples of HC below the limit	% Samples of patients below the limit	HC median, IQR [pg/ml]	POAG median, IQR [pg/ml]	OHT median, IQR [pg/ml]
IL1β	0.22	59	0	0.11, 0.11–11.66 (*n* = 39)	17.72, 14.90–32.32 (*n* = 23)	24.42, 17.89–65.08 (*n* = 8)
IL10	0.41	78	0	0.2, 0.2–0.2 (*n* = 42)	280.8, 183–322.3 (*n* = 19)	257.4; 244.1–335 (*n* = 7)
IL4	0.19	49	7	2.65, 0.1–23.11 (*n* = 37)	10.5, 5.63–12.98 (*n* = 21)	9.46, 8–11.22 (*n* = 9)
IFNγ	3.94	25	21	761.7, 79.02–1701 (*n* = 36)	507.1, 1.97–731.5 (*n* = 21)	713.3, 522.8–1127 (*n* = 8)
VEGF	0.59	12	0	555.2, 256.7–856.5 (*n* = 43)	922, 568–1030 (*n* = 20)	953.4, 738.2–1061 (*n* = 9)
MIF	0.5	66	31	0.25, 0.25–704 (*n* = 38)	578, 0.25–1063 (*n* = 21)	376.2, 29.59–816.3 (*n* = 8)

HC, healthy controls; POAG, open angle glaucoma; OHT, ocular hypertension; IQR, interquartile range; n, number of subjects available for analysis

In addition, we tested the concentration of cytokines in tears with respect to sex; the results, however, showed no differences between females and males in any of the cytokines.

### Concentration of cytokines in tears in relation to clinical characteristics 

The concentration of cytokines in tears was studied in relation to (1) intraocular pressure; (2) RNFL thickness on the basis of results from optical coherence tomography; (3) and treatment - type, duration and content of preservatives. To evaluate the effect of mentioned clinical characteristics on cytokine levels in tear fluid, we merged patients with the two different diagnoses into one group and analyzed the relationship between cytokine concentrations regardless of the diagnose. No significant differences in cytokine concentrations have been observed between patients with normal IOP (IOP lower than 20 mmHg) and patients with intraocular pressure greater than or equal to 20 mmHg. Significantly higher concentrations of IL1β, IL10 (*p* < 0.0001) and VEGF (*p* < 0.02) cytokines were observed in both, patients with IOP ≥ 20 mmHg and those with normal IOP compared to HC subjects (please see Table 3). Also, patients with IOP ≥ 20 mmHg presented with higher concentration of MIF in tears compared to HC (*p* = 0.05, please see Table 3).

**Table 3:** Median and interquartile range of analyzed cytokines in both groups of patients and healthy controls

Cytokine	HC	Patients IOP* p* < 20 mmHg	Patients IOP ≥ 20 mmHg	Patients OCT in norm	Patients OCT out	Medication without BAC	Medication with BAC
	Median, IQR	Median, IQR	Median, IQR	Median, IQR	Median, IQR	Median, IQR	Median, IQR
	[pg/ml]	[pg/ml]	[pg/ml]	[pg/ml]	[pg/ml]	[pg/ml]	[pg/ml]
IL1β	0.11, 0.11–11.66 *n* = 39	20.13, 15.12–32.32 *n* = 20	22.3, 12.9–72.66 *n* = 11	16.97, 9.87–37.58 *n* = 12	22.91, 15.12–38.14 *n* = 13	23.11, 15.94–42.41 *n* = 14	19.19, 12.79–33.93 *n* = 17
IL10	0.2, 0.2–0.2 *n* = 42	267.4, 231.6–329.8 *n* = 16	275.1, 216.8–317.2 *n* = 10	297.5, 214.8–364.9 *n* = 10	257.4, 230.1–302.8 *n* = 11	255.7, 234.1–341.4 *n* = 10	285.4, 194.8–317.4 *n* = 16
IL4	2.65, 0.1–23.11 *n* = 37	9.61, 6.38–11.22 *n* = 18	10.71, 6.35–14.49 *n* = 12	8.77, 6.01–11.4 *n* = 14	10.93, 6.96–14.69 *n* = 11	9.76, 6.22–12.55 *n* = 13	10.2, 6.55–12.01 *n* = 17
IFNγ	761.7, 79.02–1701 *n* = 36	517.7, 1.97–878.2 *n* = 18	599.7, 360.8–886.1 *n* = 12	599.7, 105.7–740.8 *n* = 12	517.7, 279.3–1273 *n* = 12	587.4, 377.1–1218 *n* = 14	549.1, 1.97–740.8 *n* = 16
VEGF	555.2, 256.7–856.5 *n* = 43	927.9, 616.3–1054 *n* = 18	950.8, 774.4–1032 *n* = 12	970.3, 582.7–1073 *n* = 12	760, 546.1–1052 *n* = 12	929.9, 546.1–1003 *n* = 12	948.2, 675.7–1086 *n* = 17
MIF	0.25, 0.25–704 *n* = 38	540, 0.25–818.1 *n* = 17	548.4, 9.36–1005 *n* = 12	531.5, 18.47–818.1 *n* = 13	686.6, 0.25–1187 *n* = 13	376.2, 0.25–916.5 *n* = 12	686.6, 18.47–916.2 *n* = 17

HC, healthy controls; IOP, intraocular pressure; BAC, benzalkoniumchloride; IQR, interquartile range; OCT, optical coherence tomography; n, number of samples with accessible characteristic

Based on the results from the OCT examination, patients were divided into 2 groups: an OCT result corresponding to the normal thickness of RNFL (designated norm) and an OCT result characteristic for lower thickness of RNFL at least in 1 quadrant (termed out). Significant differences in cytokine concentrations with respect to RNFL thickness were not detected.

The levels of studied cytokines did not differ among patients treated with different types of medication or between the groups of patients treated with medication containing or without benzalkoniumchloride (BAC). Duration of treatment did not seem to have any effect on the levels of cytokines in tears of treated patients.

### Th1 versus Th2 pathway

**Fig. 2:**
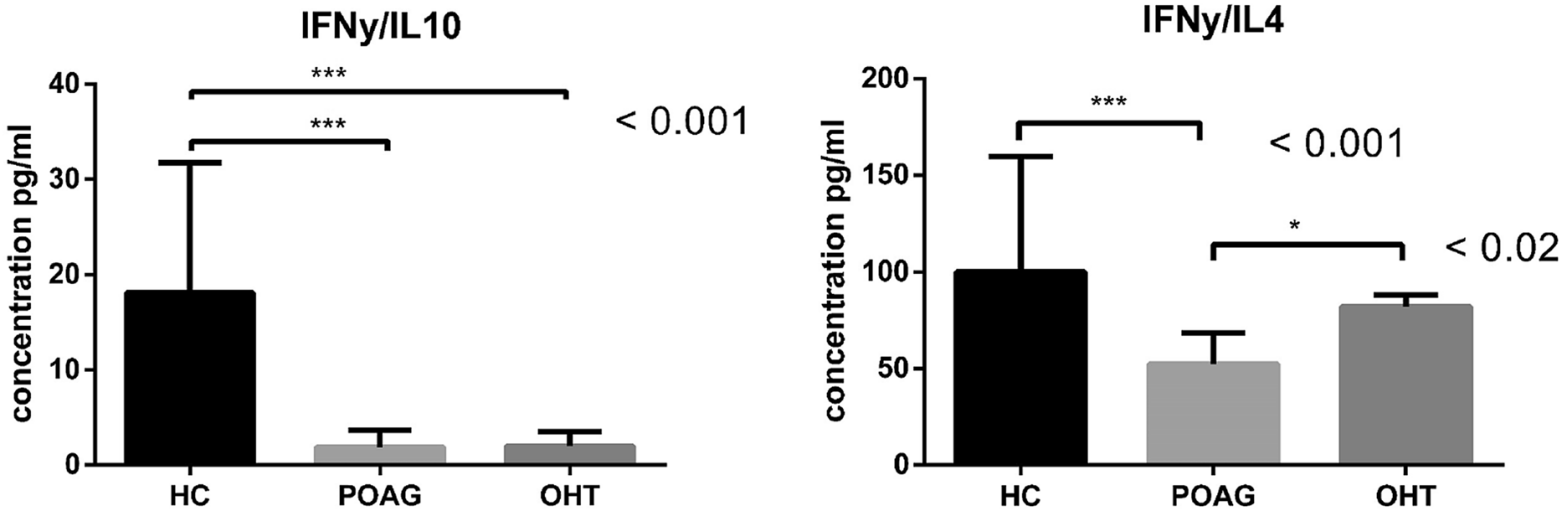
Th1/Th2 ratio in tears from healthy controls (HC), patients with glaucoma (POAG) and patients with ocular hypertension (OHT). Th1 response is represented by IFNγ cytokine; cytokines IL10 or IL4 represent Th2 response. Columns represent the median ratio of cytokines with interquartile range. Three asterisks denote statistically significant difference *p* < 0.001, one asterisk denotes statistically significant difference *p* < 0.05

To assess whether the Th1 or Th2 immune response is predominant in some of the studied groups, we included only samples where all three cytokines IFNγ, IL10 and IL4 could be detected. In patients as well as in HC, the Th1 pathway represented by IFNγ cytokine was activated to a greater extent than was Th2 path characterized by IL10 or IL4 cytokines. Nevertheless, the IFNγ/IL10 ratio was significantly lower in both POAG and OHT compared to HC (POAG: median 1.87, 1.41–3.72 (pg/ml), OHT: median 2.0, 1.83–3.53 (pg/ml), HC: median 18.12, 7.95–31.74 (pg/ml), *p* < 0.001, please see Fig. 2). The IFNγ/IL4 ratio was significantly lower in POAG in comparison with both, HC and OHT (POAG: median 52.41, 40.81–68.53 (pg/ml), OHT: median 82.26, 61.47–88.2 (pg/ml), *p* < 0.02, HC: median 100, 78.94–159.8 (pg/ml), *p* < 0.001, please see Fig. 2).

## Discussion

Tears hold an important place in cytokine research as markers of immunoinflammatory response in various eye diseases. This is supported by a result from study suggesting that elevated concentrations of certain inflammatory cytokines such as IL1β or TNFα in the aqueous humor of patients with eye disorders may diffuse from the retina to the anterior chamber where it may end up in the tear fluid through the optic-corneal button interface [19, 20]. Most studies examining the levels of cytokines in tears of glaucoma patients do not distinguish between POAG and OHT diagnoses. The attention should be drawn to the fact that there is a 9.5% probability of developing POAG within 60 months in untreated, only follow-up patients with high IOP [21]. In glaucoma, increased levels of several inflammatory cytokines have been observed in the aqueous humor and have been associated with increased death of retinal ganglion cells and elevated IOP, suggesting a plausible role in its pathogenesis [22, 23]. Thus, in the presented study, the cytokines IL1β, IL4, IL10, IFNγ, MIF and VEGF were quantified in tears derived from healthy individuals and from patients with POAG or OHT to see potential differences in inflammation on ocular surface between the two diagnoses. Cytokines were further studied depending on the magnitude of intraocular pressure and the results of the OCT examination in patients to evaluate the effect of glaucoma and various clinical factors on the cytokine composition of tears. Nevertheless, though the OHT patients in our study were characterized as follow-up, untreated subjects, their ocular tension on the day of tear collection was under the 23 mmHg (mean 20 mmHg). This is why patients in the OHT group have not needed treatment with IOP-lowering drugs so far.

For each cytokine, the detection limit was determined under which it was impossible to quantify the cytokine. In healthy controls, an appreciable number of samples below the detection limit could be found for each cytokine. The highest number of samples below the detection limit showed for the IL10, IL1β, IL4 and MIF cytokines. Thus, in some healthy individuals certain cytokines appear to be in tears at very low concentrations or not even produced at all. This assumption is confirmed by other studies showing that not all cytokines are detectable in tears from healthy individuals [24, 25]. Specifically, analysis of 30 different cytokines in tears using Luminex method revealed 5 cytokines undetectable at all and another 12 cytokines that were detected in tears from only some of the healthy subjects examined [25]. Inflammatory cytokines can be undetectable not only in tears from healthy individuals, but also in their sera, for example pro-inflammatory IL1β [24]. Accordingly, in our study, this cytokine belongs to the least detectable markers in healthy controls tears (detected only in 41% of samples). Different situation was noticed in patients: using the Bio-Plex method, only cytokines IL4, MIF and IFNγ could not be detected in all patient samples; the percentage of samples below the detection limit, however, has never exceeded 31%.

Currently, direct comparison and evaluation of data published elsewhere are impossible due to the usage of different methods under diverse conditions, quantification of various cytokines, as well as for the variable methods of collecting tears. This is also supported by the comparison of our data with results from the study where the authors used the same method: the magnitude lower values are presented compared to our results on 5 of 6 studied cytokines [26], possibly indicating a more pronounced pro-inflammatory condition on the eye surface of subjects involved in GRIM study.

The assumption of ocular surface inflammation is confirmed by the statistically significant higher concentrations of IL1β, IL10 and VEGF in tears of eye-ill people, and additionally, significantly higher concentration of MIF cytokine in tears derived from POAG patients. In contrast to these results, the Th1/Th2 ratio is significantly lower in POAG and OHT compared to HC. These differences may be due to the fact that IL1β, IL10, IL4 and MIF are undetectable in the tears of the majority of healthy subjects, while they are detectable in all or nearly all patient samples.

Most studies examine cytokine levels in long-term treated patients with ocular disorders; it is known that long-term antiglaucoma treatment is accompanied by higher concentrations of inflammatory cytokines in the aqueous humor in comparison to cytokine levels in the aqueous humor of patients without treatment [27]. Based on this finding, one could expect long-term treated patients to have elevated cytokine concentrations in tears compared to untreated patients, but the opposite may prove to be true: As far as we know, the presented data indicate for the first time that inflammation is present also in untreated patients with ocular hypertension. Moreover, by the significantly higher IFNγ/IL4 ratio we demonstrate even stronger inflammation on the ocular surface of the untreated follow-up OHT subjects compared to treated POAG patients, finding further supported by insignificantly higher concentrations of IL1β and IFNγ in tears of untreated patients with OHT compared to long-term treated patients with POAG. The question remains whether inflammation on the eye surface in POAG patients is caused by the disease *per se* or can be attributed to long-term treatment with preservative eye drops. Nevertheless, since the presented study does not include glaucoma patients without treatment or ocular hypertension patients who are receiving treatment, this perturbing issue could not be elucidated here.

Focusing on treatment in more details, the presence of the preservative agent benzalkoniumchloride in some of the drugs may represent agent influencing the cytokine composition of tears. Several studies indicate that there are differences between patients treated with BAC-containing and BAC-free drops [28–30]; in our study, however, only a trend toward stronger inflammation on the eye surface can be noticed in patients treated with BAC-conserved drugs compared to patients treated with BAC-free drops. On top of that, this output can be affected by the number of drops applied per day and therefore, the conclusions are not so clear. The outcome is consistent with a finding of a higher concentration of 22 cytokines, though non-significant, and a significant increase in the concentration of other 4 cytokines in tears of patients treated with preserved latanoprost compared to patients treated with preservative-free latanoprost [31]. Contrary to preservatives, different types of medication do not seem to affect the inflammation on the eye surface in a different manner [32]. In the presented study, though, the POAG group was quite homogeneous with respect to medication – ﻿most patients were treated with prostaglandin analogs – and this finding could not be verified.

The magnitude of intraocular pressure and the thickness of the retinal nervous fiber layer represent factors with so far uncertain effect on the condition of the ocular surface. Experimental elevation of IOP in glaucoma rat models is known to affect the expression of mRNA in retinal cells, which is associated with neuroinflammatory response and cellular apoptosis [33]. As mentioned earlier, several inflammatory cytokines in the aqueous humor of POAG patients have been found to be associated with increased retinal ganglion cell death and elevated IOP [22, 23]. The link between ocular pressure and tear cytokine levels also shows a study by Geoffrion et al. [20] that indicates an association between an IOP elevation and an increase in levels of IFNy and IL1β in tear fluid. In our study, however, the level of IOP does not seem to affect the composition of tears between the group of patients with normal IOP and IOP higher than 20 mmHg. Our results show only higher concentration of IL1β, IL10, VEGF, MIF in tears of both patient groups or one of the patient groups compared to HC. Our results may reflect small difference in IOP levels in patient groups (IOP* p* < 20 mmHg, mean: 15 mmHg; IOP ≥ 20 mmHg, mean: 22 mmHg). Similarly, when we studied the correlation between the thickness of RNFL and the concentration of cytokines in tears, we did not see any significant results. In contrast to the factor of treatment, which may act directly on the surface of the eye, the ophthalmological factors as IOP and RNFL may act more on the expression of cytokines in the retina than on the expression of cytokines by cells on the eye surface. Therefore, further investigation in larger cohorts of patients homogeneous in treatment but with more profound differences in the level of intraocular pressure and RNFL thickness is necessary.

We detected and quantified 6 cytokines in tears of POAG and OHT patients, some of which were examined in tears of glaucoma patients for the very first time. Based on presented results we conclude: (1) The eye surface of patients with treated POAG and untreated OHT shows an inflammatory environment compared to healthy controls (significant differences in levels of IL1β, IL10, VEGF, MIF); (2) Although both diseased groups have been diagnosed with their disease for a long time, there is a trend to higher levels of inflammatory cytokines IL1β and IFNγ and significantly higher IFNγ/IL4 ratio in tears of patients with untreated OHT compared to patients with long-term POAG; (3) Relationship between inflammatory/anti-inflammatory cytokines in tears of long-term POAG or OHT patients and clinical characteristics, magnitude of IOP and thickness of RNFL has not been confirmed. The limitation of the study is a number of patients, particularly in the OHT group, and the lack of OHT patients on treatment and POAG subjects without treatment, which would allow us to answer more clearly the question of how much the disease itself can affect the composition of tears on the surface of the eye. This should be taken into an account when interpreting the data and also in future studies.

Understanding how cytokines function on the surface of the eye with regard to various eye diagnoses, risk factors, types of treatment, intraocular pressure, changes in the optic nerve, and other clinical parameters may lead to a determination of the appropriate, accurate, and specific treatment for an individual POAG patient, or to early detection of conversion of OHT to glaucoma. This should lead to a reduction in ocular surface inflammation which is shown in our study and by others, and to a subject´s better condition without feeling of dry or irritated eyes.

## Data Availability

The datasets are available from the corresponding author on reasonable request.
